# Editorial: Epigenomic drivers of cancer disparities

**DOI:** 10.3389/fonc.2024.1387049

**Published:** 2024-04-04

**Authors:** Luciane R. Cavalli, Rachel E. Ellsworth, Ritu Aneja

**Affiliations:** ^1^ Research Institute Pelé Pequeno Príncipe, Faculdades Pequeno Príncipe, Curitiba, PR, Brazil; ^2^ Department of Oncology, Georgetown Lombardi Comprehensive Cancer Center, Georgetown University, Washington, DC, United States; ^3^ Department of Department of Nutrition Sciences, School of Health Professions, University of Birmingham at Alabama, Bethesda, MD, United States; ^4^ Murtha Cancer Center/ Research Program, Uniformed Services University of the Health Sciences and Walter Reed National, Birmingham, AL, United States

**Keywords:** cancer health disparities, minority (diverse) populations, epigenomic diversity, social epigenomics, epigenomic deregulation, epigenomic profiling

Multiple non-biological and biological factors contribute to cancer health disparities, which are shaped by racism and social inequalities. Although environmental exposures, social stressors, and lifestyle are established risk factors for cancer, their contributions to racial disparities in cancer incidence, outcomes, and mortality remain unclear.

Epigenomic modifications bridge biological and social determinants of health, potentially influencing cancer development and progression. Advancements in multi-omics molecular profiling, tumor categorization, and early diagnosis have led to reduced cancer mortality. However, epigenomic profiling data from ethnically diverse populations are limited. This gap impedes a comprehensive understanding of tumor epigenomes in minority groups.

To address these limitations, this Research Topic compiles studies exploring the role of epigenomic deregulation in cancer disparities. Seven articles (four original research articles and three reviews) focusing on epigenomic deregulation in various cancer types were included in this Research Topic.

Wang et al. investigated ethnic disparities in the associations between gastric precancerous lesions (GPL) and lifestyle factors in Mongolian and Han Chinese populations. Analysis of 61,638 patients (11% of whom were Mongolians) revealed significant ethnic variations in the correlation between GPL and modifiable lifestyle factors. Notably, alcohol consumption and physical inactivity were strongly linked to GPL development in Mongolians, whereas smoking was strongly associated with GPL development in Han Chinese individuals. Insufficient consumption of vegetables and fruits increased the risk of GPL in Mongolians but not in Han Chinese. These findings align with previous research highlighting the impact of excessive alcohol consumption on gastrointestinal cancer risk among Mongolians (1, 2) and underscore the importance of addressing high-risk lifestyle factors to reduce the development of GLP and its progression to gastric cancer in Mongolians. The study also emphasizes the role of genetic variations induced by heavy alcohol intake in the formation of reactive oxygen species and the development of gastric cancer.

In a comprehensive examination of genomic characteristics across racial groups (White Americans, Black Americans, Asians, Native Americans, Native Hawaiians, Pacific Islanders, and Hispanics/Latinos), Shi et al. shed light on the molecular alterations that may underlie disparities in clinical management and outcomes among patients with lung adenocarcinoma. Analysis of samples from 6,238 patients revealed higher rates of insertions and deletions in Native Americans, variable alterations in actionable targets (e.g., *EGFR, KRAS, ALK, RET*, and *ERBB2*) among racial groups, and distinct copy number alterations (most frequently affecting amplifications at 5p15.33, 7p11.2, 12q15, and 14q13.3 and deletions at 3p24.1, 9p21.3 and 10q21.2) in each race. However, the lack of data on socioeconomic status (SES) in this study limited a more comprehensive correlation analysis.

Accumulating evidence supports the hypothesis that SES is associated with increased cancer risk and poor cancer outcomes through epigenetic mechanisms. Nonetheless, data on the association between adverse sociodemographic characteristics, tumor methylation, and mortality are limited (3). In an interesting study, Miller-Kleinhenz et al. investigated the association between structural racism (measured as contemporary redlining) and DNA methylation in breast tumors, demonstrating that epigenetic perturbations may mediate the impact of structural racism on breast cancer mortality. Contemporary redlining involves the systematic denial of mortgage loans based on location, affecting specific racial or socioeconomic groups (4). Along with other discriminatory housing practices contributing to segregation, redlining has been linked to structural financial inequities associated with poor breast cancer survival (5, 6). Analysis of data from 80 patients uncovered associations between redlining and changes in DNA methylation in breast tissue, affecting genes associated with tumorigenesis, inflammation, and immune function. The study also demonstrated an association between contemporary redlining and accelerated epigenetic aging, highlighting the potential prognostic implications of structural racism.

A study by Xu et al. underscores that age-specific epigenetic modifications may contribute to disparities in cancer outcomes among different age groups. Epigenome-wide gene-age interaction analysis in patients with oral squamous cell carcinoma demonstrated reversed effects of *MORN1* DNA methylation on survival between young and older patients. Specifically, *MORN1* hypomethylation was associated with favorable survival in older patients, but poor survival in young patients. The authors explored the potential functions of *MORN1* and found that it was involved in several immune-related pathways. The authors also developed a prognostic model incorporating the *MORN1*-age interaction and clinical information, which showed better accuracy than the model with only clinical variables.

The association between epigenetics and age was also explored by Valencia et al. The authors demonstrated that DNA methylation-based accelerated age, as captured by epigenetic clocks, was associated with breast cancer risk. This finding underscores the importance of considering epigenetic age as a contributing factor to disparities in breast cancer incidence and outcomes. However, the lack of data on social aspects, epidemiological risk factors, and ancestrally diverse populations makes it challenging to discern the interactions between race, social environment, and methylation patterns as drivers of breast cancer disparities.

Waseem et al. showed that miR-99b-5p contributes to the aggressiveness of prostate cancer in African American patients. The authors proposed that downregulation or deletion of miR-99b-5p leads to upregulation of mTOR, androgen receptor (*AR*), and *AR* coactivator *SMARCD1*. This cascade promotes metabolic reprogramming, potentially driving the aggressiveness of prostate cancer and its progression to castration-resistant prostate cancer. Conversely, overexpression of miR-99b-5p inhibits this cascade, inducing metabolic signaling that suppresses cell proliferation and enhances apoptosis. Moreover, overexpression of miR-99b-5p renders prostate cancer cells more sensitive to the *AR* antagonist enzalutamide, suggesting a potential novel strategy for treating prostate cancer in African American patients.

Finally, Hao et al. reviewed the role of epitranscriptomics in the development, maintenance, and function of cancer stem cells, which are potent drivers of tumorigenesis and therapeutic resistance. RNA modifications may contribute to disparities in cancer development and outcomes by differentially regulating the development and function of cancer stem cells.

Collectively, these studies underscore the complex interplay between genetics, epigenetics, and socio-environmental factors in cancer health disparities. Comprehensive profiling and characterization of tumor epigenomes in ethnically diverse populations is needed to better understand the multifaceted determinants of cancer health disparities and bridge the racial gap in cancer mortality through development of targeted therapies for minority populations.

## Author contributions

LRC: Writing – original draft, Writing – review & editing. RE: Writing – review & editing. RA: Writing – review & editing.
